# Supplementary material for: Sexual health behavior, health status, and knowledge among queer womxn and trans men in Kenya: An online cross-sectional study
Source: PLoS One. 2022 Jun 17;17(6):e0268298. doi: 10.1371/journal.pone.0268298 (PMC9205489; doi:10.1371/journal.pone.0268298)
Supplement: Attachment — Submitted filename: Rebuttal letter.pdf [file pone.0268298.s001.pdf]

# Sexual health behavior, health status, and knowledge among queer womxn and trans men in Kenya:

# an online cross-sectional study

Rebuttal Letter

March 2022

Dear Reviewer,

Thank you very much for your kind words and productive feedback.

Please find below our responses.

1. In the introduction, when referring to the healthcare impact of queer womxn and trans men, authors might want to refer to queer womxn and trans men, showing that sexual orientation actually affects healthcare resource utilization and health status also in countries not criminalizing consensual same-sex activities. As such, this study is also relevant in terms of cultural change.

Thank you very much for this very relevant point. This was added on page 5, line 94 onward.

2. Table 3. Could you please add percent of participants in the new grouping column?

These have now been added, thank you.

3. By any chance, have authors investigated race/ethnicity of participants?

Unfortunately, we did not.

4. “The majority, 69.3% (n=232), had never used any form of contraception”. Do you have any corresponding figure from the general population in Kenya?

Indeed, there are data! Thank you for suggesting adding this, which was done on page 18, line 356-362.

5. I really liked the use of community influencers for sharing the survey in a country that criminalizes consensual same-sex activities. While I agree influencers play a very important part in communities for sharing reliable information (e.g., 10.1016/j.msard.2018.07.046), I also believe this should be discussed among limitations with the inclusion of a subset of the queer womxn and trans men community (e.g., in-closeted people do not belonging to the community were not reached).

Thank you, we agree with this assessment and have added this to the limitations, page 24, line 514 t0 518.

Once again, we appreciate the time you took to review our manuscript and the kindness of your feedback.

Warmest regards,
Stephanie Haase
